# The psychometric status of child language assessment tools in South Africa’s official languages

**DOI:** 10.4102/sajcd.v72i1.1132

**Published:** 2025-10-21

**Authors:** Frenette Southwood, Chelsea Brönn, Heather J. Brookes, Carmen Defty, Helena Kruger

**Affiliations:** 1 Department of General Linguistics, Faculty of Arts and Social Sciences, Stellenbosch University, Stellenbosch, South Africa; 2 Child Language Development Node (South African Centre for Digital Language Resources), Faculty of Arts and Social Sciences, Stellenbosch University, Stellenbosch, South Africa; 3 Division of Speech-Language and Hearing Therapy, Department of Health and Rehabilitation Sciences, Faculty of Medicine and Health Sciences, Stellenbosch University, Stellenbosch, South Africa

**Keywords:** child language, language assessment, assessment instruments, South Africa, African languages, instrument development, instrument adaptation

## Abstract

Despite advancements in recognising the importance of early child language development and linguistic diversity in South Africa, culturally and linguistically appropriate assessment tools remain unevenly distributed across the official languages, posing considerable challenges for researchers and speech-language therapists. This study critically reviews the psychometric status of child language assessment tools in South Africa’s official languages. The current state of tool development is analysed, evaluating language assessment tools available for clinical and research purposes in each language. Tools that assess speech sounds, phonological processing and (pre)literacy skills were excluded, as were untraceable tools reported in previous publications, and tools that do not assess language only but form part of larger developmental assessments. Considering each language version of a tool, 37 traceable language assessment tools (at least 1 per official language), in various states of readiness, are available for use with South Africans aged 0–12 years. Out of these 37 tools, 5 tools, with collectively 7 language versions, have been validated and the statistical results of the validation study published: 4 for isiZulu, 2 for English and 1 for Siswati. Despite advances in tool development, there is still a severe lack of validated standardised child language measurement tools in all official languages of South Africa.

**Contribution:**

This is the most recent critical review of the psychometric properties of tools developed for assessing South African children’s language skills, and it highlights that past and current efforts in tool development are still insufficient.

## Introduction

Children who are not faring well in acquiring their language(s) or those who present with language disorders are at risk for poor literacy acquisition, which can negatively affect educational progress (Lundberg, 2006; see Duff & Tomblin, 2018). Language assessment enables the identification of these children but requires reliable, valid language assessment tools if underdiagnosis or overdiagnosis of language problems is to be avoided (Buac & Jarzynski, 2022). Furthermore, accurate language assessment with linguistically and culturally appropriate assessment tools forms the cornerstone of effective language intervention (Pascoe & Norman, 2011).

This study considers child language assessment tool development for the official languages of South Africa (SA). Tool development during apartheid reflected the general unequal treatment of languages, with more work performed for the then only two official languages (Afrikaans and English) than for African languages (isiNdebele, isiXhosa, isiZulu, Sesotho, Sesotho sa Leboa, Setswana, Siswati, Tshivenda, Xitsonga) (see e.g. Penn, 1998). Since 1994, the government has started to redress past inequities, among others, introducing policies and initiatives to elevate the status of African languages in education, the media and public life (Sokani, 2024). However, as will be shown in this paper, research on language acquisition and the development of tools with which to measure child language skills and diagnose language problems mostly remains neglected for children of all ages, particularly in African languages. Given the linguistic demographics of the country’s population – 80.7% use a language other than Afrikaans or English as home language (Statistics South Africa, 2023) – this imbalance between the country’s languages is problematic.

There have been five reviews of language assessment tools for use with SA children: Penn’s (1998) overview and Mphahlele’s (2006) inventory were written from a speech-language therapy (SLT) perspective; Pascoe and Norman (2011) gave an overview of contextually relevant resources for SA SLTs and audiologists; Pascoe et al. (2020) did a scoping review of health resources available, and Pascoe and colleagues (2011, 2020) and Pascoe and Singh (2023) included resources for use with children and adults. Except in the Pascoe and Singh (2023) publication, the scientific readiness-for-use of the available tools was not critically evaluated.

In 1998, Penn concluded that ‘resources are lacking’ (1998, pp. 265–266). Pascoe et al. (2020), 22 years later, reported 57 resources for SA languages, a seemingly substantial improvement since 1998. However, these 57 resources are not limited to child language nor to assessment, and Pascoe and Singh (2023) listed only 16 published tools for assessing SA children’s speech or language (21 if counting all language versions). For these available tools to be of diagnostic and research value, they need to have been adapted culturally and linguistically, standardised locally and have norms that are still valid (American Psychological Association, 2017), otherwise they do not allow for benchmarking of a child’s language performance and are therefore not necessarily ready for wider use.

This study critically reviews progress in tool development for measuring the language of children up to 12 years, for research and diagnostic purposes, in SA’s official languages. Considered for review were published tools and those in development, and for each, it was indicated whether (1) it was purpose-developed, translated and/or adapted; (2) there were any pre-pilot activities to increase linguistic and cultural appropriateness; (3) tools that were a pilot study was conducted; (4) the tool had been standardised; (5) reliability and validity were tested and reported and (6) the tool had been normed. The following were excluded: tools (1) assessing speech sound and phonological development and (pre)literacy skills; (2) lacking evidence of principled linguistic and cultural adaptation – for instance, tools that were merely translated or were adapted informally and/or live (e.g., Feris, 2017); (3) that were cited in other publications but were not traceable for verification purposes; (4) devised as intervention material that could incidentally be used for informal assessment and/or (5) that do not assess language exclusively but include a language subtest when assessing child development more broadly.

## Traceable child language assessment tools for use with South African children

The development of child language assessment tools commenced during apartheid, focusing on Afrikaans and English, the then well-resourced, official languages. Original tools developed for Afrikaans by 1994 include *Afrikaanse Reseptiewe Woordeskattoets* (*ARW, ‘Afrikaans Receptive Vocabulary Test’;* Buitendag, 1994) and *Afrikaanse Semantiese Taalevalueringsmedium* (*AST*; *‘Afrikaans Semantic Language Evaluation Medium’*; Pretorius, 1989). An Afrikaans version of two tools that were originally developed elsewhere for child speakers of English were created during apartheid: *Illinois Test of Psycholinguistic Abilities* (*ITPA*; first edition: Kirk et al., 1967) was translated into and adapted for Afrikaans (Lotter, 1971); and *Peabody Picture Vocabulary Test* (*PPVT*; first edition: Dunn, 1959) was translated into Afrikaans (Alant & Beukes, 1986). Pre-democracy, SA English-speaking children were mostly assessed with tools developed elsewhere in minority-world contexts–and this practice is ongoing (cf. Van Dulm & Southwood, 2013)–or with informally adapted versions thereof (such adaptation typically entailing replacing words with their SA English equivalents), without validating or renorming the tools for SA children. Such tools are excluded from this review based mainly on exclusion criterion (2) above. When considering exclusion criteria (1) to (5) above, no tools developed during apartheid for African languages could be included in this review.

Below, an overview of tools developed for SA languages during and after apartheid is presented. A summary of these tools and their selected psychometric properties appears in Table 1 (see also Appendix 1), showing the methodological steps completed during tool development.

**TABLE 1:** Developmental stages of South African child language assessment tools.

Tool	Publication date	Translated from an existing tool?	Adapted?	Pre-piloted?	Standardised?	Acceptable reliability?	Acceptable validity?	Normed?
Illinois Test of Psycholinguistic Ability – Afrikaans	1971	Yes	Yes	Yes	No	NR	NR	No
Peabody Picture Vocabulary Test (PPVT) – Afrikaans	1986	Yes	No	Yes	No	No	Values NR	No
Afrikaans Semantiese Taalevalueringsmedium†	1989	No	No	Yes	No	Yes	Yes	Yes; *n* = 900
Language Development Survey†	1989	No	Yes	Yes	No	NR	Yes	No
Afrikaanse Reseptiewe Woordeskattoets†	1994	No	No	Yes	Yes	Yes	Yes	Yes; *n* = 900
PPVT – isiZulu	1994	Yes	Yes	Yes	No	NR	NR	No
Zulu Expressive and Receptive Language Assessment (ERLA) test (ZERLA)	1995	No	No	Yes	Yes; *n* = 188	Yes	Values NR	No
PPVT – Sesotho sa Leboa	1997	Yes	Yes	Yes	Yes; *n* = 152	NR	NR	No
Sesotho ERLA test	1997	Yes	No	No	No	NR	NR	No
Setswana ERLA test	1997	Yes	No	No	No	NR	NR	No
Tshivenda ERLA test	1997	Yes	No	No	No	NR	NR	No
Xitsonga ERLA test	1997	Yes	No	No	No	NR	NR	No
Test of Ability to Explain†	2006	Yes	Yes	Yes	No	Yes	Yes	No
LITMUS-Multilingual Assessment Instrument for Narratives (MAIN) – Afrikaans	2012	Yes‡	Yes‡	No	No	NR	Values NR	No
LITMUS-Crosslinguistic Lexical Tasks (CLT) – Afrikaans	2016	No	No	Yes	No	NR	In progress	No
LITMUS-CLT – isiXhosa	2016	No	No	Yes	No	NR	In progress	No
LITMUS-CLT – SA English	2016	No	No	Yes	No	NR	In progress	No
LITMUS-MAIN – isiXhosa	2019	Yes	Yes	No	No	NR	NR	No
LITMUS-MAIN – Tshivenda	2019	Yes	Yes	No	No	NR	NR	No
LITMUS-MAIN – isiZulu	2019	Yes	Yes	Yes	No	NR	Values NR	No
Ingwavuma Receptive Vocabulary Test†	2020	No	Yes	Yes	No	Yes	Yes	No
Receptive and Expressive One-Word Picture Vocabulary Test	2021	Yes	Yes	No	No	Yes	NR	No
Sentence Repetition Test	2022	No	No	Yes	No	NR	NR	No
Productive Vocabulary Test†	2022	No	No	No	Yes; *n* = 412	Yes	Yes	No
MacArthur-Bates Communicative Development Inventory (CDI) – all 11 spoken official languages	2025	Yes	Yes	Yes	No	Yes	Values not yet available	In progress
Diagnostic Evaluation of Language Variation (DELV) – Afrikaans	In progress	Yes	Yes	Yes	No	NR	Values not yet available	In progress; *n* = 1000
DELV – SA English	In progress	No	Yes	Yes	No	NR	Values not yet available	In progress; *n* = 500

Note: Please see full reference list of this article, https://doi.org/10.4102/sajcd.v72i1.1132, for more information.

NR, not reported; LITMUS, Language Impairment Testing in Multilingual Settings; SA, South Africa.

† , Tool has been validated;

‡ , The leader of the team developing the Afrikaans version of *LITMUS-MAIN* (i.e., Daleen Klop) was however part of the original, 2012 team developing the protocol for rendering various culturally appropriate language versions of this tool (see Gagarina et al., 2012).

For the SA language versions of the following 10 tools that were included in the list in Table 1, either (1) no reliability, validity or norming study was conducted; (2) reliability and/or validity was studied but the obtained statistical values were not reported, and therefore reliability and validity could not be verified or (3) the norming study is dated:

For vocabulary or semantics: The translation of the *PPVT* (first edition: Dunn, 1959) for Afrikaans overseen by Alant and Beukes (1986); translation and adaptation of the *PPVT* for isiZulu (Naidoo, 1994); and the translation and adaptation of *PPVT-Revised* for Sesotho sa Leboa (Pakendorf & Alant, 1997); *AST* (Pretorius, 1989) and *ARW* (Buitendag, 1994). The last two were developed more than 30 years ago. Given that the lexico-semantics of languages change over time, reconsideration of the linguistic and visual content of these tools is indicated; therefore, they are not necessarily currently suitable for diagnostic purposes. *Language Impairment Testing in Multilingual Settings (LITMUS)-Crosslinguistic Lexical Tasks* (*CLT*; cf. Haman et al., 2015) was developed for Afrikaans, isiXhosa, and SA English (cf. Potgieter & Southwood, 2016) using the same protocol for each language version, to increase comparability across languages in terms of item difficulty. For each language version, three mother-tongue speakers provided general information on approximately 1000 words and on the appropriateness of the picture accompanying each word. From these, 165 clearly depictable words were selected, and three mother-tongue speakers provided information on the phonetic, morphological, semantic and etymological characteristics of each. These were used to calculate a complexity score. The latter was considered together with the reported age of acquisition (of 20 adults per language) to calculate each word’s complexity index. Items were selected based on their complexity index. *CLTs* were piloted with 26 Afrikaans-, 34 English- and 10 isiXhosa-speaking children. Amendments were made to the tool, and larger-scale data collection for validation purposes is underway. Each 120-item tool assesses the comprehension and production of nouns and verbs based on pictures and is meant for use with 3- to 5-year-olds.

For morphosyntax: Palmer’s (2022) sentence repetition test, the only tool in SA Sign Language considered for this review, consists of 20 sentences (simple, moderate or complex, based on their estimated grammatical complexity). It was piloted with 40 Deaf children aged 7 to 9 years. Scores increased with an increase in child age and an increase in length of exposure to SA Sign Language.

For narration: *LITMUS-Multilingual Assessment Instrument for Narratives* (*LITMUS-MAIN*; cf. Gagarina et al., 2012) has an Afrikaans version by Klop and Visser (cf. Gagarina et al., 2019a); isiXhosa version (Klop & colleagues, 2019; cf. Gagarina et al., 2019b), Tshivenda version (Mabaso & Tshikonelo, 2019) and isiZulu version (Ndlovu et al. cited in Gagarina et al., 2019c; Ndlovu & Klop, 2023 – piloted with 28 children aged 9 to 11 years). *LITMUS-MAIN* allows for assessment of multilingual children’s understanding and production of narratives in two or more languages using picture-based stories presented through different elicitation modes (model story, telling, retelling). Stimulus material is controlled to be (1) equivalent in terms of cultural appropriateness and cognitive and linguistic complexity (see Klop & Visser, 2020; Ndlovu & Klop, 2023), and (2) parallel in macro- and microstructure within and across language versions (see Gagarina et al., 2012). For all language versions of *LITMUS-MAIN*, aspects of content validity were addressed by adapting the picture stimuli for cross-cultural appropriateness.

For tools assessing more than one language domain: *ITPA*, originally designed to measure spoken and written language abilities of English-speaking children (Hammil et al., 2001), was preliminary translated into and adapted for Afrikaans (Lotter, 1971); *South African Language Assessments* (*SALA*; Bortz, 1997) for Setswana, Sesotho, Tshivenda and Xitsonga, viz. the translated versions of *Zulu Expressive and Receptive Language Assessment* test (*ZERLA*; discussed below) and the Afrikaans and SA English *Diagnostic Evaluation of Language Variation* (*DELV*; Seymour et al., 2015) of which the development is ongoing (cf. Van Dulm & Southwood, 2008). *DELV* is not yet ready for clinical use but has stimulated some research (cf., e.g., Southwood 2013; Southwood & White, 2021a, 2021b). It is a picture-based tool assessing the comprehension and production of various aspects of syntax, pragmatics, semantics and phonology in children aged 4 to 9 years. The Afrikaans and SA versions were linguistically and culturally adapted in conjunction with SLTs who spoke non-mainstream varieties of these languages. The Afrikaans version was piloted among 48 children aged 5 to 9 years, all speakers of the so-called Kaaps variety of Afrikaans, and their *DELV* scores correlated well with their *ARW* scores (Southwood & Van Dulm, 2009). Norming is underway and nearing completion. However, given that norming commenced in 2008, some data points might need replacement before norm calculation (F. Southwood, personal communication).

Table 2 shows the sample sizes and statistical values of the tools listed in Table 1 for which the statistical analyses of the reliability, validity and/or norming studies were reported, barring the *ARW* and *AST* for reasons mentioned above. The content of Table 2 is discussed further.

**TABLE 2:** Available reliability, validity and norming statistics.

Tool	Reliability	Validity	Norming
Language Development Survey†	Reliability NR	Good; significant at 5% level	No
Zulu Expressive and Receptive Language Assessment	Poor test-retest reliability (correlation coefficient −0.27) Good mark-remark reliability (correlation coefficient −0.76)	Values NR	No
Test of Ability to Explain†	Good inter-scorer reliability (*r* = 0.96) Good internal consistency or reliability (correlation coefficient 0.79)	Good content validity (correlation of 0.31–0.64)	No
Ingwavuma Receptive Vocabulary Test†	Good§ internal consistency and reliability (Cronbach’s alpha: *α* = 0.74) Good test-retest reliability (+0.8)	Fair overall sensitivity (66.7%) Poor overall specificity (33%)	No
Receptive and Expressive One-Word Picture Vocabulary Test	Good test-retest reliability (Pearson’s correlation coefficient of 1)	Validity NR	No
Productive Vocabulary Test†	Good: Split-half reliability English (*α* = 0.85, 95% CI = [0.68, 0.90]), isiZulu and Siswati (*α* = 0.76, 95% [CI = 0.64, 0.82]); Spearman-Brown split-half coefficients English (0.91) isiZulu and Siswati (0.79)	Good internal validity (*α* = 0.76, 95% CI = [0.64, 0.82]) for both isiZulu and Siswati	No
MacArthur-Bates Communicative Development Inventory (CDI) – all 11 spoken official languages	Poor to good across language versions (0.216–0.711)	Validation study in progress	Commencing 2025

Note: Reliability classification: Good = tool internal consistency > 0.80, test-retest or mark-remark reliability > 0.70, validity deemed high (without necessarily specifying what that is; depends on how closely related the compared scales are) (Aaron Kaat, personal communication). Fair = 35% – 60%. Poor < 35%.

NR, not reported; CI, confidence interval.

† , tool has proven validity.

### Valid tools assessing lexicon and semantics

*Language Development Survey* (*LDS*; Rescorla, 1989), a parent-report screening tool consisting of a vocabulary checklist of 309 words in 14 semantic categories used to screen for language delay in 2-year-olds, was originally developed in the United States for child speakers of American English. This preliminary English version was adapted for use with SA children by a panel of teachers, SLTs, augmentative and alternative communication specialists, an occupational therapist and a mother of a child with language impairment, after which it was trialled with five parents. Subsequently, 22 items were replaced with culturally and linguistically appropriate items, and this updated version was trialled with 40 parents of typically developing toddlers from middle-class backgrounds who spoke isiZulu, English, Afrikaans, Sepedi, Siswati or Setswana (Gonasillan et al., 2013). *LDS* results correlated ‘with [other] research results on early acquisition of vocabulary’ (Gonasillan et al., 2013, p. 13), and there were significant correlations between the scores of the American and SA toddler groups for 10 of the 14 categories.

Mazibuko and Chimbari (2020) developed the *Ingwavuma Receptive Vocabulary Test* (*IRVT*) as a screening and diagnostic tool for use with isiZulu-speaking 4- to 6-year-olds. This one-word picture vocabulary test assesses comprehension of nouns, verbs, adjectives and other categories. *Ingwavuma Receptive Vocabulary Test* was culturally and linguistically adapted from, among others, the *British Picture Vocabulary Scale* (Dunn, 2009) and then piloted with 10 isiZulu-speaking pre-schoolers (exact ages not specified) and five PhD students for the development of the initial blueprint of the tool. To finalise the vocabulary list, community research assistants, SLTs and preschool teachers were also consulted. *Ingwavuma Receptive Vocabulary Test* showed good internal consistency and reliability, good test-retest reliability (*n* = 8) and fair overall sensitivity with the final version of the tool having been administered to 51 children aged 4 to 6 years.

*Receptive and Expressive One-Word Picture Vocabulary Test* was adapted by Jordaan et al. (2021) from *Receptive One-Word Picture Vocabulary Test 4* (Martin & Brownell, 2011a) and *Expressive One-Word Picture Vocabulary Test 4* (Martin & Brownell, 2011b) to assess the receptive, expressive and conceptual vocabulary of bilingual Grade 1 and Grade 2 learners (isiZulu-English, Afrikaans-English). The tool assessed the ability to identify objects, actions and concepts (*Receptive Vocabulary* subtest) and to name objects, actions and concepts (*Expressive Vocabulary* subtest). The tool was translated into and adapted for isiZulu and Afrikaans and then piloted with 120 children: 30 bilingual English-isiZulu speakers, 30 bilingual English-Afrikaans speakers and 60 monolingual English speakers. The tool showed good test-retest reliability.

*Productive Vocabulary Test isiZulu/Siswati* and *Productive Vocabulary Test English* (Wilsenach & Schaefer, 2022) were developed by selecting words from the *British National Corpus/Corpus of Contemporary American English* (*BNC/COCA)* word list (Nation, 2017) that were deemed culturally appropriate and translating them into the relevant local language, sourcing culturally appropriate images to accompany these words. The tools were piloted in rural areas with 20 Grade 3 participants (10 Siswati- and 10 isiZulu-speaking), of which 13 completed both language versions. A study among 412 children showed that the (1) English and (2) isiZulu and Siswati versions had good split-half reliability, and both the isiZulu and Siswati had good internal validity.

### Valid tools assessing verbal problem-solving

*Test of Ability to Explain* was developed to measure the verbal problem-solving skills of rural isiZulu-speaking 7- to 12-year-olds (Solarsh & Alant, 2006). It was translated and adapted from the *Test of Problem Solving* (Zachman et al., 1984), whereafter the pre-pilot version was trialled with six children (an 8-, 10- and 13-year-old of each sex). It was then piloted with 60 children, 20 of each of the above-mentioned ages. Solarsh and Alant (2006) report good inter-scorer reliability and good content validity.

### Valid tools assessing several language domains

*Zulu Expressive and Receptive Language Assessment (ZERLA)*, developed by Bortz (1995) for 2- to 7-year-olds, was designed based on the Soweto variety of isiZulu to assess receptive and expressive morphosyntactic and semantic abilities (noun class system, agreement and verb structure, relatives and passives). The preliminary version was administered to 20 children aged 2 to 5 years in three separate pilot studies (*n* = 60), after which the amended version was administered to 255 children aged 2 to 5 years (Bortz, 1995). Items were reduced from 148 to 90, and the scoring method was changed. The tool was then administered to 303 children aged 3 to 4 years (Bortz, 1995). Bortz (1995) reported good internal consistency or reliability and good mark-remark reliability. In a supplementary study in the pre-standardisation phase, *ZERLA* was able to differentiate between 17 children with and 19 children without language impairment (Bortz, 1995; values not reported). In addition to the diagnostic version of *ZERLA*, there is a screening version containing items that demonstrated both good discrimination and appropriate difficulty during item analysis (Bortz, 1997).

*MacArthur-Bates Communicative Development Inventory* (*CDI*; Fenson et al., 2007) is a parent-report tool measuring gestures, word comprehension and word production in 8- to 18-month-olds (*CDI-Words&Gestures*); and word and grammar production in 16- to 30-month-olds (*CDI-Words&Sentences*). Originally developed for American English, *CDIs* have been adapted for over 100 languages (https://mb-cdi.stanford.edu) including all spoken official languages of SA (Brookes et al., 2025). Teams of language experts adapted the American English *CDI* based on their own language knowledge, spontaneous samples of children’s languages and data from previous research on child language acquisition in the respective languages (Brookes et al., 2025). For each language, *CDIs* were refined through focus group discussions with professionals and parents, piloted with 20 infants and 20 toddlers and standardised with 100 infants and 100 toddlers, producing final long-form versions. The results indicate reliability through correlations between age and vocabulary size (Brookes et al., 2025). Validation in terms of criterion-related concurrent validity is in progress, and norming will commence in 2025, starting with Afrikaans and isiXhosa (H. Brookes, personal communication).

### Summary

Tools for which either reliability and validity statistics are available or for which validation is underway appear in Table 3, per language. Of these tools, only *LDS* was available pre-1994.

**TABLE 3:** Child language assessment tools, per official language (only tools with reported reliability or validity statistics for at least one of their language versions or tools being validated).

Tool	Afrikaans	isiNdebele	isiXhosa	isiZulu	Sesotho	Sesotho sa Leboa	Setswana	SiSwati	SA English	SA Sign Language	Tshivenda	Xitsonga
Communicative Development Inventories	•	•	•	•	•	•	•	•	•	-	•	•
Ingwavuma Receptive Vocabulary Test†	-	-	-	•	-	-	-	-	-	-	-	-
Language Development Survey	-	-	-	-	-	-	-	-	•	-	-	-
Literacy Impairment Testing in Multilingual Settings-Crosslinguistic Lexical Tasks	•	-	•	-	-	-	-	-	•	-	-	-
Productive Vocabulary Test†	-	-	-	•	-			•	•	-	-	-
Receptive and Expressive One-Word Picture Vocabulary Test	•	-	-	•	-	-		-	•	-	-	-
Test of Ability to Explain†	-	-	-	•	-	-	-	-		-	-	-
Zulu Expressive and Receptive Language Assessment	-	-	-	•	-	-	-	-		-	-	-

SA, South Africa.

† , Acceptable validity.

## Discussion and conclusion

Since the onset of democracy, 12 child language tools have been added to the small number of pre-1994 tools in SA’s now-official languages (see Table 1), covering the age range from infancy to 12 years. Of these, six are translations and/or adaptations of tools developed elsewhere (*CDI, DELV, all but the Afrikaans version of the LITMUS-MAIN, PPVT, Receptive and Expressive One-Word Picture Vocabulary Test, Test of Ability to Explain*) and six were originally developed by SA researchers (*IRVT, LITMUS-CLT, Productive Vocabulary Test, SALA, Sentence repetition test, ZERLA)*. Eleven of these post-1994 tools are available in at least one of SA’s non-West-Germanic official languages, with *DELV* being available only in Afrikaans and English. When each language version is counted separately and when *LDS* (a validated, pre-democracy tool) is added, 39 language assessment tools, in varying states of psychometric readiness, are available for use with SA children (at least one per official language).

However, this calculation presents an overly positive picture. In total, when excluding tools that need norming or renorming, there are four validated tools for use with SA children: *IRVT, LDS, Productive Vocabulary Test* and *Test of Ability to Explain*. These collectively have six language versions, with *LDS* being available in English only. The language for which most validated tools are available is isiZulu (3), followed by English (2) and Siswati (1). Based on the above, some progress has been made in language assessment tool development post-1994; however, there are no currently normed tools available. (Norming of two tools–*DELV*, and the Afrikaans and isiXhosa versions of *CDI*–is about to commence; H. Brookes & F. Southwood, personal communication). Scientifically sound child language assessment tools are unevenly spread across languages. This, coupled with insufficient support for research in indigenous languages, is disconcerting: tool development needs to be theory-driven and evidence-based if tools are to be valid (APA, 2017), which means that they need to be grounded in the findings of robust research into the acquisition of SA’s languages by child speakers of a wide range of ages. Such research is emerging slowly. Furthermore, children growing up multilingually, which includes many children in SA, need to be assessed in each of their languages (see, e.g., Buac & Jarzynski, 2022), which requires appropriate assessment tools in those languages. This adds gravity to the general unavailability of SA child language assessment tools: not only is there a need for tools to be developed to completion, but multiple language versions of these tools are also needed. In addition, the fact that SA languages have different varieties requires tool developers to specify the geographic area(s) from which their participants were sampled in order to allow the end-users of a tool to evaluate the applicability of the tool for the child(ren) to whom they wish to administer it.

To address the lack of valid and standardised measurement tools, the research methodologies used during tool development may require critical evaluation, as more community co-design may be needed, which could be well served by adopting participatory action research. Furthermore, larger teams of experts in child language development from different disciplines, as well as well-trained multilingual research teams, are needed to conduct large-sample studies in SA languages to validate and standardise tools. Data from large studies will also contribute to improving existing theories in the field of child language acquisition that have been built on findings predominantly generated in minority-world contexts. Scientifically sound child language research embedded in local child language socialisation practices will allow for accurate child language assessment. This is more than a diagnosis- or research-related ideal; it is a moral obligation. Assessment tools that do not consider linguistic and cultural variation and do not attempt to avoid linguistic and cultural bias cannot deliver accurate assessment results (cf. Pascoe & Norman, 2011), and without accurate assessment, child language intervention flounders. Conversely, scientifically sound, contextually appropriate early childhood interventions can lead to SA children gaining a strong language foundation on which to build literacy skills.

## Appendix 1

### Child language assessment tools referred to and/or critiqued

Alant, E., & Beukes, S. (1986). The application of the Peabody Picture Vocabulary Test-revised (PPVT-R) to non-mainstream children. *South African Journal of Communication Disorders, 33*(1), 7–14. https://doi.org/10.4102/sajcd.v33i1.318

Bortz, M. (1995). *A language assessment for preschool Zulu speaking children*. Master’s thesis, University of the Witwatersrand. Retrived from http://hdl.handle.net/10539/20880

Bortz, M. (1997). *South African language assessments manual*. Stass Publications.

Buitendag, M.M. (1994). *Afrikaanse Reseptiewe Woordeskattoets* [*Afrikaans Receptive Vocabulary Test*]. Human Sciences Research Council.

Dunn, L.M. (1959). *Peabody picture vocabulary test*. American Guidance Service.

Dunn, L.M. (2009). *The British picture vocabulary scale*. GL Assessment Limited.

Fenson, L., Marchman, V.A., Thal, D.J., Dale, P.S., Reznick, J.S., & Bates, E. (2007). *MacArthur–Bates communicative development inventories user’s guide and technical manual*. Paul H. Brookes.

Gagarina, N., Klop, D., Kunnari, S., Tantele, K., Valimaa, T., Balciuniene, I., Bohnacker, U., & Walters, J. (2012). MAIN: Multilingual assessment instrument for narratives. *Zentrum für Allgemeine Sprachwissenschaft Papers in Linguistics, 63*, 20. https://doi.org/10.21248/zaspil.63.2019.516

Gagarina, N., Klop, D., Kunnari, S., Tantele, K., Välimaa, T., Bohnacker, U., & Walters, J. (2019a). MAIN: Multilingual assessment instrument for narratives – Revised. Materials for use. *Zentrum für Allgemeine Sprachwissenschaft Papers in Linguistics, 63*, 20

Gagarina, N., Klop, D., Kunnari, S., Tantele, K., Välimaa, T., Bohnacker, U., & Walters, J. (2019b). MAIN: Multilingual assessment instrument for narratives – Revised. Materials for use. *Zentrum für Allgemeine Sprachwissenschaft Papers in Linguistics, 63*, 20.

Gagarina, N., Klop, D., Kunnari, S., Tantele, K., Välimaa, T., Bohnacker, U., & Walters, J. (2019c). Multilingual assessment instrument for narratives – Revised. Materials for use. *Zentrum für Allgemeine Sprachwissenschaft Papers in Linguistics, 63*, 20.

Gonasillan, A.S., Bornman, J., & Harty, M. (2013). Vocabulary used by ethno-linguistically diverse South African toddlers: A parent report using the language development survey. *South African Journal of Communication Disorders, 60*(1), 10–15. https://doi.org/10.4102/sajcd.v60i1.4

Haman, E., Luniewska, M., & Pomiechowska, B. (2015). Designing Cross-Linguistic Lexical Tasks (CLTs) for bilingual preschool children. In S. Armon-Lotem, J. De Jong, & N. Meir (Eds.), *Assessing multilingual children: Disentangling bilingualism from language impairment* (pp. 196–240). Multilingual Matters.

Hammil, D.D., Mather, N., & Roberts, R. (2001). *Illinois test of psycholinguistic abilities* (3rd ed.). PRO-ED.

Kirk, S.A., McCarthy, J., & Kirk, W.D. (1967). *The Illinois test of psycholinguistic abilities*. University of Illinois Press.

Klop, D., & Visser, M. (2019). MAIN: Multilingual assessment instrument for narratives – Revised. Materials for use. Afrikaans version. *Zentrum für Allgemeine Sprachwissenschaft Papers in Linguistics, 63*, 20.

Mabaso, N.P., & Tshikonelo, N.V. (2019). Tshivenḓa version of the multilingual assessment instrument for narratives – Revised. In N. Gagarina, D. Klop, S. Kunnari, K. Tantele, T. Välimaa, U., & J. Walters (Ed.), *MAIN: Multilingual Assessment Instrument for Narratives – Revised. Materials for use. Zentrum für Allgemeine Sprachwissenschaft Papers in Linguistics, 63*.

Martin, N., & Brownell, M.A. (2011a). *Expressive one-word picture vocabulary test* (4th ed.). Academic Therapy Publications.

Martin, N., & Brownell, M.A. (2011b). *Receptive one-word picture vocabulary test* (4th ed.). Academic Therapy Publications.

Mazibuko, X., & Chimbari, M. (2020). Development and evaluation of the Ingwavuma receptive vocabulary test: A tool for assessing receptive vocabulary in isiZulu-speaking preschool children. *South African Journal of Communication Disorders, 67*(1), 1–10. https://doi.org/10.4102/sajcd.v67i1.780

Naidoo, P. (1994). *Test translation in a South African context using the Peabody picture vocabulary test –Revised*. Master’s thesis, University of Durban Westville.

Nation, P. (2017). *The BNC/COCA Level 6-word family lists (Version 1.0.0)*. Victoria University of Wellington. Retrived from https://www.wgtn.ac.nz/lals/resources/paul-nations-resources/vocabulary-lists

Pakendorf, C., & Alant, E. (1997). Culturally valid assessment tools: Northern Sotho translation of the Peabody Picture Vocabulary Test-Revised. *South African Journal of Communication Disorders, 44*, 3–12. https://doi.org/10.4102/sajcd.v44i1.223

Palmer, A. (2022). *Developing a sentence repetition test for the evaluation of deaf children’s use of South African Sign Language*. Master’s thesis, Stellenbosch University.

Pretorius, A. (1989). *Die Afrikaanse Semantiese Taalevalueringsmedium*. Pretorius.

Seymour, H.N., Roeper, T.W., & De Villiers, J. (2005). *Diagnostic evaluation of language variation: Norm referenced*. Pearson.

Sokani, A. (2024). Government efforts and shortcomings in elevating indigenous languages in South Africa over 30 years: A systematic review. *International Journal of Research in Business and Social Science (2147- 4478), 13*(10), 145–153. https://doi.org/10.20525/ijrbs.v13i10.3594

Solarsh, B., & Alant, E. (2006). The challenge of cross-cultural assessment – The test of ability to explain for Zulu-speaking children. *Journal of Communication Disorders, 39*, 109–138. https://doi.org/10.1016/j.jcomdis.2005.11.002

Zachman, L., Jorgenson, C., Huisingh, R., & Barrett, M. (1984). *Test of Problem Solving (TOPS)*. Lingui Systems.
